# Supplementary material for: Are carbon emissions trading and green financial instruments synergistic? -Comprehensive quantitative research based on content analysis
Source: PLoS One. 2024 Mar 7;19(3):e0298601. doi: 10.1371/journal.pone.0298601 (PMC10919601; doi:10.1371/journal.pone.0298601)
Supplement: S1 Data — (ZIP) [file pone.0298601.s001.zip › Data/地级市Z_F工作报告.zip!/║╙▒▒/╩»╝╥╫»╩╨/└┤╫╘╣1⁄2╓┌║┼í╛┬φ┐╦╩2╛▌═°í┐_2019.docx]

各位代表：

现在，我代表石家庄市人民政府，向大会作政府工作报告，请予审议，并请各位市政协委员和列席人员提出意见。

一、2018年工作回顾

过去的一年，我们深入学习贯彻习近平新时代中国特色社会主义思想和党的十九大精神，树牢“四个意识”，坚定“四个自信”，做到“两个维护”，在省委、省政府和市委的正确领导下，紧紧依靠全市人民，坚持稳中求进工作总基调，践行新发展理念，把握高质量发展要求，深化供给侧结构性改革，统筹做好稳增长、促改革、调结构、治污染、惠民生、防风险各项工作，扎实开展“双创双服”“双问计”活动，较好完成了市十四届人大三次会议确定的各项目标任务，开创了现代省会、经济强市建设新局面。

——经济运行稳中有进，发展质量效益持续提升。全市生产总值增长7.5%。一般公共预算收入、全部财政收入分别完成519.7亿元、1076.3亿元，增长12.8%、13.6%，顺利实现“跨五越千”。减税降费117.3亿元。固定资产投资增长6.4%，社会消费品零售总额增长9.1%。主要经济指标增速均好于全省。规上高新技术产业增加值增长17.3%，连续8年实现两位数增长。

——产业结构稳中调优，“4+4”现代产业格局基本确立。三次产业结构优化为6.9∶37.6∶55.5，服务业对经济增长的贡献率超过70%。落实“4+4”现代产业发展决策部署，出台“1+8”行动方案，1788个“4+4”现代产业项目完成投资占全市固定资产投资的58.3%，对投资的贡献率达到65.6%。“4+4”现代产业税收增长14.6%，成为引领石家庄高质量发展的主要力量。

——城市建设稳中提质，省会综合竞争力大幅提高。积极创建国家卫生城市，顺利通过全国文明城市国家复检，省级以上园林县城实现全覆盖。成功举办首届国际数字经济博览会，打造了省会又一张靓丽名片，成为数博会永久举办地。成功承办省运会、省残运会，连续举办了中国国际通用航空博览会、石家庄国际投资贸易洽谈会、市旅游产业发展大会等高水平会展活动，被评为“中国会展名城”“亚太旅游目的地城市”，省会的影响力、吸引力、辐射力明显提升。

——生态环境稳中向好，空气质量持续改善。单位生产总值能耗降低率、化学需氧量、二氧化硫、氨氮、氮氧化物排放量完成省下达的目标任务。全市地表水达到或优于省考断面的比例提高10个百分点。森林覆盖率达到40.6％。全市PM2.5平均浓度下降16.3％，综合指数下降率为13.6%，首次实现全年“零爆表”。全长42公里的滹沱河生态修复工程全面开工建设，城区段生态景观长廊正式向市民开放。

——民生事业稳步前行，人民群众福祉显著增强。城乡居民人均可支配收入分别增长8%左右、8.5%左右。城镇新增就业19.5万人，农村转移劳动力5.2万人，城镇登记失业率3.3%。纳入省考核的490个“三难”房地产遗留问题全部解决。完成老旧供热管网改造358公里，提前一周带热试运行，供热效果明显提高。高考成绩再创历史新高，勇夺全省文科、理科成绩“双第一”，职教园区一期6所学校全部入驻。在全国主要城市中，交通拥堵率降幅排名第二，社会治安名列第三，食品安全位列第八。10件利民惠民实事顺利完成。民之所望，政之所向。石家庄，正在用民生温度彰显城市发展的新高度。

一年来，面对错综复杂的外部环境，面对产业转型、结构调整、污染治理的艰巨任务，我们凝心聚力，积极应对，战胜各种困难挑战，推动各项工作迈上新台阶。

（一）抓转型、转动能，着力构建“4+4”现代产业格局。强化产业发展的顶层设计，研究制定支持“4+4”现代产业发展的系列政策文件。新一代信息技术产业快速发展，与中电科13所和54所签署战略合作框架协议，通信与导航设备、新型显示、应用电子和软件等优势产业加速集聚，主营业务收入增速突破20%。生物医药健康产业稳步增长，“全国心脑血管医药产业知名品牌创建示范区”正式获批筹建，以岭药业“理临科产教”五位一体发展模式成为全国中医药科技成果产业化的创举，生物医药健康产业税收增长17.3%。先进装备制造产业竞争力明显增强，中渥电气成套设备生产基地等重点项目相继开工，奇瑞新能源汽车首车下线。现代商贸物流业提档升级，华润万象城、北国奥特莱斯水世界、天山海世界二期建成开业，掀起省会新消费热潮。金融创新开发区建设初见成效，金融业增加值增长8.1%，税收增长22.5%。全域旅游开创新局面，旅游业总收入增长25.8%。科技服务与文化创意产业大幅增长，节能环保产业规模不断壮大，实现利税分别增长26%、30%。

开发区成为“4+4”现代产业发展的重要平台，全年主营业务收入增长20%以上。大力推进传统产业改造升级，组织实施“千企转型”行动，134个技改项目列入省“千项技改项目”。县域经济活跃度明显提升，长安区、藁城区跻身全国综合实力百强区，正定县、井陉矿区入围全国投资潜力百强县区。重点龙头企业发展实现历史性跨越，石炼化公司利税超百亿，君乐宝乳业集团主营业务收入超百亿，石药集团成为香港恒生指数成份股中首支蓝筹医药股。

（二）抓改革、促开放，着力增强经济发展活力。深化供给侧结构性改革，坚定不移去产能，全年压减水泥产能357.5万吨、焦炭130万吨，圆满完成年度目标任务。持续深化“放管服”改革，取消下放行政权力事项76项，在全省率先建成“互联网+政务服务”平台，实现“五十二证合一”，企业开办时间压减至2.5个工作日，新增市场主体19.4万户。金融改革措施落地见效，全年新增贷款1148.9亿元，小微企业贷款风险补偿机制向全国推广。创新土地管理工作，全面推行“先做土地、后做项目”和大片区土地收储做法，全市新增占补平衡指标4万亩。旗帜鲜明支持民营经济发展，制定实施促进民营经济高质量发展的系列政策，敬业集团、东旭集团、天山集团跻身全国民营企业500强。招商引资成效明显，一批优质企业和项目签约落地，全球领先的生物医药研发企业药明康德、技术世界领先的现代生物制药企业沃森生物开工建设，全国成立最早、规模最大、连接全球创客资源网络最紧密的柴火创客空间引进运营。对外开放不断扩大，主动融入“一带一路”建设，中欧（石家庄）班列正式开通，打通了通往欧洲市场的贸易大通道；积极落实京津冀协同发展战略，全力支持雄安新区规划建设和冬奥会筹办；加强对外交往，80多个国家驻华大使、参赞到石家庄共谋发展。全市实际利用外资14.9亿美元，增长7.5%；进出口总值完成864.1亿元，增长8.6%。

（三）抓创新、建体系，着力打造区域发展新高地。坚持把创新作为引领高质量发展的第一动力，深入实施创新驱动发展战略，落实配套政策措施，完善科技创新生态体系。诺贝尔奖获得者工作站达到6家，院士工作站达到64家。认定工业企业研发机构764家，新认定国家企业技术中心3家，国内唯一的“平板显示国家工程实验室”去年在我市建成并通过验收。省级以上孵化器、加速器和众创空间达到78家。新增国家级高新技术企业515家、达到1315家，新增科技型中小企业2027家、达到11132家，均居全省第一。新增专利授权8334件，技术合同成交额完成95亿元。君乐宝乳业集团获“中国质量奖提名奖”，东旭集团获“国家科技进步一等奖”，我市成为国家知识产权示范城市。强化创新人才队伍建设，在全国省会城市中第一个出台《人才发展促进条例》，发放“人才绿卡”3580张，柔性引进诺贝尔奖获得者6名、两院院士24名，培养引进国家“千人计划”“万人计划”入选者和长江学者17名，引进世界500强大学和国家“双一流”建设高校毕业生3800多名，为高质量发展提供了智力支撑。

（四）抓规划、提品质，着力提升城市建设管理水平。坚持现代化标准，紧紧扭住城市规划设计、基础设施建设、精细化管理等环节，推进城市高质量发展。高起点修编新一轮城市总规，形成阶段性成果。中央商务区开发建设正式启动，车辆厂和百年广场临建全部拆除。地铁全年运送旅客8700余万人次，1号线二期实现“洞通”。南二环东西延、和平路高架桥西延、和平路中华大街立交桥全线贯通，联石丰道路拓宽工程除个别节点外基本贯通，中华大街南延、建设大街南延等13条“断头路”彻底打通。太行山高速石家庄段主体完工通车，津石、石衡高速开工建设。新建地下综合管廊20公里。新购置水洗机扫车239辆，主城区道路机扫率达到90%。新建停车泊位2800多个，智慧泊车被评为全国公共停车场运营管理示范项目。新建改造西兆通等公园7座，建成街旁游园7处，新增绿地面积938万平方米。国家新型城镇化综合试点任务基本完成，常住人口城镇化率达到63.83%。

（五）抓基础、补短板，着力推进乡村全面振兴。调整优化农业产业结构，大力发展科技农业、绿色农业、品牌农业、质量农业。继续稳定农业综合生产能力，粮食、蔬菜及食用菌、肉蛋奶总产量分别达到424.8、502.5、187.9万吨。农业科技装备水平得到新提升，主要农作物耕种收机械化水平达到97.3%。农产品质量持续提高，建成农业标准化生产基地110个，新认证“三品一标”企业79家、达到353家。智慧农业大数据平台投入使用，农产品二维码质量追溯平台实现农业县（市、区）全覆盖。深入开展农村人居环境整治行动，新改建农村公路436公里，天然气入户率达到44.8%，光纤覆盖率达到99%，无害化卫生厕所普及率提高到70%，农村生活垃圾处理长效机制实现全覆盖。成功打造井陉古村落等6个美丽乡村旅游度假区，晋州周家庄入选中国美丽休闲乡村。农村集体产权制度改革扎实推进，完成3979个村集体资产清产核资，成功获批国家整市推进试点。

（六）抓重点、攻难点，着力加强生态环境综合治理。践行“绿水青山就是金山银山”的理念，扎实做好中央和省环保督查“回头看”问题整改，污染防治攻坚战取得新进展。空气质量持续改善，围绕退“倒十”目标，坚持科学治污、精准治污、铁腕治污、协同治污，聘请国家级专家团队跟踪加密研判，设立会商指挥平台统筹协调调度，实行周会商、月调度，采取超常规措施，深入开展“两散两车三高三尘”综合治理，取得阶段性成效。完成农村“双代”取暖改造31.9万户，任务完成量全省第一。全面取消散煤，市域内35蒸吨以下燃煤锅炉实现“清零”，压减燃煤120万吨。巩固深化“散乱污”企业整治和涉VOCS企业治理成果。修复责任主体灭失矿山1652亩，治理黄土裸露地面107万平方米。PM2.5平均浓度从2016年的99微克/立方米下降到72微克/立方米，下降浓度绝对值在全国169个重点城市中排名第一。积极开展水土污染防治，严格落实“河长制”，土壤污染治理与修复试点工作有序推进，造林绿化73万亩。环境执法力度持续加大，立案处罚4527件。全市上下勠力同心、众志成城，付出了巨大努力，圆满完成了省定目标任务，让人民群众享受到更多的蓝天白云、绿水青山。

（七）抓民生、办实事，着力增强人民群众获得感幸福感安全感。持续加大民生投入，民生事业支出759.3亿元，占一般公共预算支出的76.3%。精准脱贫成效显著，5.7万人稳定脱贫，226个贫困村出列，平山县正式脱贫摘帽，赞皇县、灵寿县、行唐县达到脱贫出列标准。社会保障水平继续提高，发放援企稳岗补贴1.77亿元，企业退休人员养老金人均月增133.3元，城乡低保标准分别提高到每人每月610元、每人每年4400元；建成棚户区改造住房2.8万套，改造提升老旧小区210个，建成公租房7473套，分配入住比例达到97.2%，超额完成省定任务。社会事业协调发展，新建普惠性幼儿园44所，消除义务教育大班额1861个，我市普职融通改革获得“全国教育改革创新特别奖”，石家庄外国语教育集团荣获“全国脱贫攻坚组织创新奖”。市中医院东院区建成投用，新增国家级“群众满意乡镇卫生院”20个，建成社区首席专家工作室104个，新增医养结合机构44个，成功创建“全国基层中医药工作先进市”。市委党校新校区基本建成，市图书馆被评为“国家一级馆”。建成全民健身路径100条，实施农村体育健身工程100个。社会治理不断创新，深入开展“扫黑除恶”专项斗争，持续推进风险排查化解活动，深化安全隐患整治，社会大局保持和谐稳定。

一年来，我们坚决落实全面从严治党战略部署，大力推进政府自身建设，认真做好中央、省委巡视反馈意见整改，严格执行八项规定及其实施细则。加强法治政府建设，自觉接受人大法律监督、政协民主监督和社会监督，定期向市人大常委会报告工作、向市政协通报情况，354件人大代表建议、512件政协委员提案全部按时办复。服务型政府建设取得新成效，“双创双服”重点帮扶企业和项目反映的问题得到较好解决，“双问计”争取上级政策199项、各类项目7359个。国防动员和“双拥共建”深入开展，民族宗教、人民防空、气象地震、妇女儿童、老龄、残疾人等事业都有了新的发展和进步。

各位代表，回顾过去的一年，我们经历了迎接挑战、抢抓机遇的不平凡历程，也收获了推进高质量发展的丰硕成果，全市稳的态势在持续、进的力度在加大、好的因素在累积、惠的成效在显现。实践中，我们深化了对做好新形势下经济工作的认识。一是讲政治，坚定方向。坚定不移以习近平新时代中国特色社会主义思想统领各项工作，在市委的正确领导下，全力推动党中央、国务院和省委、省政府重大决策部署，第一时间在省会落实落地。二是讲统筹，科学发展。运用马克思主义方法论，抓主要矛盾、抓矛盾的主要方面，既全面推进又重点突破，既注重当前更着眼长远，综合考量、精准施策，牢牢把握经济工作主动权。三是讲规则，厘清边界。遵从经济规律、尊重市场规则，厘清并恪守政府、市场、社会三方边界，充分发挥市场在资源配置中的决定性作用和更好发挥政府作用。四是讲法治，履职尽责。弘扬宪法精神，坚持依法行政，自觉尊法、学法、守法、用法、护法，用法治思维推动工作开展，用法治手段破解矛盾难题，用法治规范保障发展成果。五是讲担当，干事创业。始终保持奋发有为的精神状态，把推进落实作为职责之要，真抓实干，层层传导责任、明确时限要求、加强督导检查，善做善成，久久为功。

各位代表！事非经过不知难，成绩来之不易。这是以习近平同志为核心的党中央引航定向的结果，是省委、省政府关心支持的结果，是市委正确领导的结果，是全市人民团结一心、砥砺奋进的结果。在此，我代表市人民政府，向全市人民，向人大代表、政协委员，向各民主党派、工商联、无党派人士和人民团体，向驻石人民解放军、武警官兵和政法干警，向中直、省直驻石单位，向所有关心支持石家庄改革发展的港澳台同胞、海外侨胞和国际友人，致以崇高的敬意和衷心的感谢！

在肯定成绩的同时我们也清醒看到，当前我市发展中还存在不少困难和问题。一是经济发展质量效益不够高，工业经济低位运行，战略性新兴产业还未形成有效支撑，实体经济发展依然面临不少困难。二是创新能力仍需加强，基础研究投入不够，高新技术企业、研发机构与发达地区差距较大，人才储备还不能满足高质量发展的需要。三是环境治理任务艰巨，环境容量趋于饱和，大气、水、土壤污染治理需要付出更多更大努力。四是城市现代化水平有待提高，基础设施历史欠账较多，城市形象和品位需进一步提升。五是营商环境不优、干部作风不严不实等问题不同程度存在。对此，我们必须增强忧患意识，正视困难差距，下大力气加以解决。

二、2019年工作总体要求

今年是新中国成立70周年，是全面建成小康社会、实现第一个百年奋斗目标的关键之年。当前，世界面临百年未有之大变局，全球产业链加速重组，经济结构加快重塑，经济增长动能转弱，贸易保护主义重新抬头，经济治理体系变革处于重要时期，国际协作共赢局面发生深刻变化。国内经济运行稳中有变、变中有忧，经济面临下行压力，困难和挑战有所增多。但更要善于从长期大势认识当前形势，经济发展长期向好的基本面没有变，我国发展仍处于并将长期处于重要战略机遇期。深刻审视面临的新形势、新任务，新时代的石家庄正处在一个大有可为的战略机遇期、乘势跃升的攻坚突破期和担当使命的接续奋斗期，我们要坚决贯彻中央“巩固、增强、提升、畅通”八字方针，认真落实省委、省政府的决策部署，紧紧围绕市委十届六次全会确定的目标任务，进一步夯实发展基础、释放发展活力、集聚发展势能、提高发展质量，努力开创省会高质量发展的美好未来。

今年政府工作的总体要求是：坚持以习近平新时代中国特色社会主义思想为指导，全面贯彻党的十九大和十九届二中、三中全会精神，认真落实省委九届八次全会和市委十届六次全会精神，坚持稳中求进工作总基调，坚持新发展理念，坚持推动高质量发展，坚持以供给侧结构性改革为主线，坚持深化市场化改革、扩大高水平开放，加快构建“4+4”现代产业发展格局，继续打好三大攻坚战，全面实施城市建设攻坚提质，大力推进乡村振兴战略，加强保障和改善民生，加快建设现代省会、经济强市，为全面建成小康社会收官打下决定性基础，以优异成绩庆祝中华人民共和国成立70周年。

2019年主要预期目标：地区生产总值增长7%左右，一般公共预算收入增长7.5%左右，规模以上工业增加值增长4%以上，服务业增加值增长10%以上，固定资产投资增长6%左右，社会消费品零售总额增长9.5%，城乡居民人均可支配收入均增长7.5%左右，居民消费价格涨幅控制在3.5%左右。上述目标的设定，充分考虑了我市发展的支撑条件和外部环境，统筹了总量增长和结构优化，体现了稳中求进、进中趋优的要求和高质量发展的导向。

落实总体要求，实现工作目标，我们必须坚持党对经济工作的领导，学懂弄通做实习近平新时代中国特色社会主义思想，坚决落实中央和省决策部署，坚决落实市委安排部署，坚决做到政令畅通、执行有力，知行合一、令行禁止。必须始终扭住发展第一要务，坚持以经济建设为中心，把握战略机遇期新内涵，深化经济发展规律性认识，稳就业、稳金融、稳外贸、稳外资、稳投资、稳预期，不断巩固经济持续向好态势。必须坚定不移推动高质量发展，迈改革开放之步、走创新驱动之路，激发微观主体活力，汇聚国内外优质资源，优结构增效益转动能，为高质量发展蓄能增势。必须坚持以人民为中心的发展理念，不忘初心、牢记使命，顺应民心、致力民生，在治理污染、修复生态中加快营造良好人居环境，在脱贫攻坚、推进共享中努力提高人民生活水平。只要我们紧盯发展方向、紧扣发展脉搏、紧抓发展机遇、紧跟发展步伐，就一定能够推动省会发展创造新的优势、实现新的跨越。

三、2019年重点任务

做好今年工作，必须认真贯彻党中央、国务院决策部署，全面落实省委九届八次全会和市委十届六次全会要求，坚定信心、同向发力，确保圆满完成各项目标任务。重点抓好以下九个方面：

（一）坚定不移夯实经济高质量发展基础，加快优化“4+4”现代产业发展格局。产业兴、经济强。坚持产业立市不动摇，紧紧围绕构建“4+4”现代产业发展格局，认真落实系列三年行动计划和配套措施，不断增强产业竞争力。

大力发展先进制造业。制造业是兴国之器、强国之基。立足我市产业基础和优势，聚焦“4+4”现代产业中的新一代信息技术、生物医药、装备制造、节能环保等先进制造业，找准突破点和爆发点，每个行业打造1-2个在全国乃至世界有影响的企业，推动制造业向价值链高端迈进。按照“延链”的要求，工业设计优化一批、科技创新转化一批、产业延伸集聚一批，加快推动产业链条双向延伸融合，促进制造企业由生产环节向服务环节延伸、服务企业向制造环节延长，强化制造业与现代服务业深度融合，推动工业生产提级增速。充分发挥河北工业设计创新中心作用，支持引导企业在产品研发、外观设计、产品包装等环节植入工业设计元素，促进制造业向创意、研发、售后等产业链两端延伸，不断提升工业产品的品牌效应和附加值。

大力发展战略性新兴产业。着眼产业链的关键核心、缺失环节，以“补链”为引领，培育壮大卫星导航、光电显示、新能源汽车等战略性新兴产业。细化落实省十大主攻方向、六大工程部署安排，打造一批战略性新兴产业示范基地。适应互联网经济从上半场转到下半场的新趋势，推动消费互联网向产业互联网、“互联网+”向“+互联网”转换延伸，促进产业链和价值链重构。加快5G商用步伐，加强人工智能、工业互联网、物联网等新型基础设施建设，提升企业生产要素数据化水平，提升企业运营流程数字化协同水平，实现生产要素精细化、运营流程准配化，推进经济演变和提高全要素生产效率。提前布局数字金融、区块链运用、量子通信、IT和BT的融合、生物技术等未来产业，在新一轮发展竞争中抢占先机、赢得主动。

大力发展现代服务业。支持服务业扩容提质，不断提升现代服务业对经济增长的贡献率。培育壮大金融产业，加快中央商务区、金融创新开发区建设，积极引进金融、科技、结算中心等高端企业，形成产业聚集、资本放大效应，高效服务实体经济发展。做大做强旅游业，突出生产生活生态融合，整合旅游资源，开发特色旅游，办好省、市旅游产业发展大会，打造一批旅游康养示范基地，推进全域旅游发展，全年旅游业总收入增长21%以上。加快发展现代商贸物流和会展经济，建设全国现代商贸物流中心城市；充分利用交通资源和区位优势，调整优化物流产业布局，推进综合性智能化物流基地建设，巩固提升全国物流节点城市地位。谋划筹备金融博览会，继续办好中国国际通用航空博览会、国际数字经济博览会等大型展会，全年举办各类会展活动180场次以上。

大力改造提升传统产业。以“强链”为目标，聚焦化工、食品、纺织等传统优势产业，深入实施“千企转型”活动，全面推动工业企业转向创新驱动、绿色发展、现代管理。实施新一轮技术改造和设备更新，对传统产业进行智能化、绿色化、高端化改造，滚动实施100项以上重点技改项目。加强产业链协同配套，引导中小企业向集群化、协作化转型。制定实施特色产业振兴计划，明确“一县一业”“一业一策”建设路线图，支持平山钢铁、晋州纺织、元氏化工、深泽日化、无极皮革、行唐奶业等加快转型提质，实现从关联企业集聚向个性品牌成长、再向集群品牌提升的梯次迈进，打造高层次、升级版的世界级特色产业集群，培育“专精特新”隐形冠军。

（二）坚定不移增加有效需求，努力保持经济平稳增长。稳是大局，进是趋势。顺应壮大国内市场新要求，用足用好国家积极的财政政策和稳健的货币政策，充分发挥消费的基础性作用、投资的关键性作用，支撑经济稳中求进。

全面提升消费水平。消费需求是最终需求，其规模扩大、质量提升将对产品和服务供给起到引领和倍数作用。适应消费升级趋势，增供给与优环境并举，扩规模与提水平并重，加快发展教育培训、家政服务、健康养老、文化旅游、休闲体育等服务业，积极培育定制消费、信息消费、智能消费、共享消费、体验消费等新兴消费热点，支持传统商贸企业发展线下体验、线上消费新零售模式。继续实施质量强市和标准化战略，深入推进增品种、提品质、创品牌“三品”行动。开拓农村消费市场，实施农产品电子商务等现代商贸物流工程，健全农村流通网络体系和售后体系，让老百姓吃的放心、穿的顺心、用的舒心。

全面提升投资质量。我市现阶段投资需求潜力巨大，关键是选准领域和项目，抓重点、强弱项、补短板，以精准有效投资催生优质供给，以高端高质项目引领转型升级。围绕重大项目落地投产，开展“项目落地年”行动，签约项目抓开工，在建项目抓进度，竣工项目抓投产，前期项目抓推进，加大城乡基础设施、生态环境保护、重大产业项目等领域补短板力度，推进340个省市重点项目建设，完成年度投资830亿元左右。稳步推进石钢搬迁等一批重大转型升级项目，扩大东旭玻璃基板、志诚永华新型液晶材料等一批重大科技成果产业化规模。实施精准招商，转变招商理念，强化市级统筹，解决无序低效竞争、产业同质化等问题；创新招商方式，实施产业链招商、资源补缺式招商、成果转化式招商，由引资为主转向引资、引智并重，加快招引一批投资规模大、科技含量高、税收贡献好、带动能力强的高端高新项目。

全面提升开发区能级水平。实施开发区提档升级行动，加快“新九通一平”建设，鼓励高新区、石家庄经开区朝着主导产业鲜明、产业链条完整、产城深度融合的方向发展，省级园区朝着特色突出、要素集聚、配套完善的方向发展。支持鹿泉经济开发区等条件成熟的开发区向国家级升格，加快循环化工园区转型升级步伐，推动中俄“两国双园”、鹿泉中芬产业园等中外合作产业园区建设。统筹协调、有序推进开发区托管整合，实现优势互补，完善产业布局，全面提升开发区发展水平。全市省级以上开发区主营业务收入继续保持20%以上增长。

为顺利推进“项目落地年”行动，继续实行领导干部包联重点项目制度，持续抓好市直部门与县（市、区）联合招商、捆绑考核，充分发挥考核“指挥棒”的作用，项目考核从注重开工率，向注重竣工投产、亩均效益转变；招商引资考核从注重签约率，向注重资金到位率、项目开工率转变；开发区考核从注重规模和速度，向注重质量、结构和贡献率转变，形成鲜明的政策导向，为高质量发展提供有力保障。

（三）坚定不移全面深化改革扩大开放，不断增强高质量发展的动力活力。改革不停顿，开放不止步。切实推动重大改革任务举措落地，加快形成全方位、多层次、宽领域的全面开放新格局，全力推动新时代改革开放再出发。

更大力度推进重点领域改革。深化“放管服”改革，扩大“最多跑一次”覆盖面，推进“双随机、一公开”监管，加快推动政务服务“一网通办”，年内工程建设项目审批时间压缩至70个工作日以内。深化国资国企改革，加快建立国资监管全覆盖的管理体制，设立市属产权综合交易服务机构。深化财税金融改革，全面实施预算绩效管理，规范政府举债融资机制；强化地方金融监管和服务能力，改进和增强中小微企业金融服务，引导金融业回归服务实体经济本源。深化科技体制改革，创新科技评价制度，推进科研经费使用管理、科技成果转化收益分配、科研人员薪酬制度等改革，形成有效的创新激励机制。深化综合行政执法改革，将执法力量下沉到基层，有效解决“看得见的管不了，管得了的看不见”问题。

更大力度扩大高水平对外开放。开放是机遇，开放是活力，高水平开放是高质量发展的必然选择。统筹利用国际国内两个市场、两种资源，依托正定海关、鹿泉海关、机场海关，加快发展综合保税区、海关特殊监管区，申报建立进境商品指定口岸，推进国家跨境电子商务综合试验区有关政策在省会落地。年内国际邮件互换局竣工运营。深度融入“一带一路”建设，积极开展国际产能合作，扩大先进技术设备、关键零部件和紧缺资源型产品的进口，鼓励钢铁、化工、水泥、纺织等企业“走出去”，建立境外生产加工基地。力争全年进出口总值、实际利用外资均增长5%以上。

更大力度融入京津冀协同发展。坚决落实省委、省政府纵深推进“三件大事”的决策部署，积极推动交通、生态、产业三个重点领域的深度合作，在服务对接京津雄中加快发展自己。提升交通互联互通水平，大力推进雄石城际铁路、津石高速公路等基础设施项目建设。加强产业对接协作，落实好常态化沟通机制，提高承接能力，创优承接环境，加快建设正定新区重点承接平台，吸引京津雄优质项目和科技成果落地转化，形成“京津雄研发、石家庄转化、石家庄制造”的合作模式，打造高水平协同发展示范区。其他县（市、区）发挥比较优势，高水平规划建设，努力成为承接非首都功能疏解和产业转移的“微中心”；将支持雄安新区大规模建设，转化成为加快发展自己的机遇，瞄准需求、占领市场，提供产品、壮大产业。

（四）坚定不移实施创新驱动发展战略，加快打造国家创新型城市。抓创新就是抓发展，谋创新就是谋未来。紧紧抓住石保廊全面创新改革试验区建设的机遇，加强创新要素集聚和科技成果转化，加快形成以创新为主要引领和支撑的高质量发展模式。

更加注重创新体系建设。支持高新区由生产型向研发型升级、由功能单一的产业区向产城融合的城市新区转型，打造国家自主创新示范区。发挥诺贝尔奖获得者工作站等科技平台作用，加快科技成果转化和产业加速器建设，构建开放、协同、高效的共性研发平台，健全需求为导向、企业为主体的产学研用一体化创新机制。加强科技大市场的运营管理，力争年技术合同交易额突破百亿元。全年新增科技企业孵化器4家，众创空间15家，认定市级以上技术创新中心、重点实验室、产业技术研究院35家，构筑创新创业新高地。

更加注重创新主体培育。严格执行高新技术企业所得税优惠、企业研发费用加计扣除、研发投入普惠性奖励等政策措施，支持企业建设高水平研发机构。大力实施高科技企业培育工程，建立科技型中小企业、高新技术企业、“瞪羚”企业、“独角兽”企业培育库，加大服务支持、资源配置和政策倾斜力度，年内新增国家级高新技术企业200家，培育认定科技型中小企业1300家。设立创新基金，加大研发投入，全社会研发经费支出占GDP比重达到2.5%。

更加注重军民融合发展。推动军民融合重大科技攻关，主动对接军队高端科技创新资源和成果，积极构建军民融合协同创新体系。面向航空航天、新材料、量子通信等前沿领域，发挥好河北省军民两用技术交易中心、军民融合知识产权交易中心的平台优势，深化与中电科13所、54所等国家级重点科研单位的合作，支持鹿泉加快鹿北军民融合产业新区建设，打造军民融合产业集群。

更加注重创新人才引领。实施“领头雁工程”，加强各行业高端人才引进力度。深入开展“院士专家行”活动，新增院士工作站5家。与国内一流大学、教育机构等合作建立研究生院、职业教育学院、培训中心，增强高层次人才和实用型技术人才培养能力。对创新人才、创业人才、科技人才，我们就是要给政策、给待遇，提供人尽其才、追梦圆梦的广阔舞台，让石家庄成为创新的蓝海、创业的热土、创客的乐园。

（五）坚定不移优化营商环境，毫不动摇支持民营经济发展。民营经济作用不可或缺，民营企业前途不可限量。认真贯彻习近平总书记在民营企业家座谈会上的重要讲话精神，抓好民营企业“百千万”提升工程，扶持壮大一批主业突出、核心竞争力强的民营骨干企业。

切实减轻企业税费负担。实施降本减负专项行动，进一步清理规范涉企收费，巩固行政性“零收费”成果，重拳治理收过头税和“三乱”行为，实质性降低民营企业负担。对小微企业和科技型初创企业实施普惠性优惠政策，鼓励中小微企业利用股权投资、风险投资等方式融资。抓好纾困基金管理运行，帮助生产经营正常、暂时困难的企业渡过难关。

切实提供优质高效服务。认真落实国家和省、市支持民营经济发展的各项政策措施，坚决清理阻碍政策落地的“中梗阻”问题，打通政策落实“最后一公里”。坚决打破“卷帘门”“玻璃门”“旋转门”，清除各类隐性壁垒，在经营许可、经营运行、招投标等方面一视同仁。严格落实领导干部包联责任制，建立重点企业“市长直通车”制度，为企业发展送上“石家庄版”的“政策服务包”。深入开展“双创双服”“千人帮千企”等活动，主动与民营企业家打交道、交朋友、解难题。

切实维护企业合法权益。依法保护企业家人身财产安全，完善诚信体系建设，加强知识产权保护，严厉打击侵害民营企业权益的违法行为和黑恶势力，激发和保护企业家精神，在全社会营造尊重企业家的良好氛围。各级政府都是企业的“服务员”，凡是民营企业的合理诉求，我们一定有求必应；凡是政府作出的承诺，我们一定坚决兑现。

（六）坚定不移增强辐射带动能力，努力建设现代化区域中心城市。城优旺业，城美聚才。优化城市布局，完善城市功能，提升形象品位，不断提高省会美誉度和吸引力。

实施规划设计提标行动。继续完善城市总规成果，做好滹沱河沿线、解放大街沿线、工业遗址等重点区域的城市设计。超前规划管控轨道交通沿线土地利用和产业布局。加强城市建筑风貌管控，对城市公共空间、建筑群组关系、建筑景观、建筑色彩、建筑体量等要素，进行全过程精细化管理。加快编制城市地下空间利用专项规划，统筹协调地下、地面、地上空间配置、功能衔接。严格“三条红线”划定，建立“城市体检”制度，坚持多规合一，一张蓝图绘到底。

实施城乡发展提档行动。继续实施主城区水、电、暖、气、路“五网同治”，加快推进联石丰道路贯通工程、解放大街东半幅工程等城区路网建设。充分利用人防工程等地下空间闲置资源，配建市政设施和公共服务设施。强力推进公交都市示范城市创建，确保通过国家验收。坚定不移推动北跨发展，用足用好市场机制建设正定新区，加快完善酒店、学校、医院等配套设施，实现人口、商业、产业快速集聚，打造全市发展新的增长极。推动县城建设争先进位，巩固扩大“两违”拆除成果，深入开展“园林城、卫生城、洁净城、文明城”创建活动，大手笔、高标准建设各具特色的美丽县城，年内力争正定、晋州创建成国家园林城市，元氏、平山、赞皇、赵县创建成省级洁净城市，所有县在全省排名中名次前移，更多县进入全省前30名。

实施重点工程提速行动。把建设中央商务区作为全市的“1号工程”，强力推进手续审批、土地征迁等各方面工作，年内取得明显进展。加快推进轨道交通在建工程，确保上半年1号线二期工程载客运营，2号线一期和3号线一期两边段按时间节点加紧建设，积极跑办后续规划报批工作。加快功能性场馆建设，确保城市馆5月1日前开馆，市图书馆、市档案馆按期竣工投用。推进石衡、南绕城高速公路等建设，完善对外大通道路网体系。

实施城市风貌提质行动。着力改善人居环境，深入开展老旧小区整治和棚户区改造三年攻坚行动，积极稳妥实施城中村改造，强力推进停车场建设，加快地下管廊试点和海绵城市建设，配套完善社区服务设施，让老百姓生活更加舒适便捷。

大力提升城市景观，加强城市绿化、美化、亮化、净化，高标准实施南二环东西延、和平路西延、中华大街南延等道路绿化，加大开花、彩叶、冬绿植物种植力度，推动城市绿化多彩化、艺术化。力争西环公园5月1日前完工，铁路文化公园年底前开放。大力实施街道两侧建筑外立面整治、夜景亮化完善提升工程，使城市更加靓丽多彩。

实施城市管理提效行动。城市发展三分建七分管。坚持以国家卫生城市创建为抓手，巩固和扩大全国文明城市成果，让城市更加整洁有序。强化精细管理，加强小街小巷综合整治，清理占道经营，推广垃圾分类投放，提高水洗机扫率，提升城市管理水平。强化房地产市场监管，巩固整治成果，构建长效机制，确保房地产市场健康发展。加快城市综合管理信息平台建设，实现感知、分析、服务“三位一体”城市管理，打造新型智慧城市。加强土地管控，坚持政府主导、市场化运作，节约集约用地，提升管理水平。

（七）坚定不移实施乡村振兴战略，扎实推动农业农村工作迈上新台阶。乡村振兴，产业兴旺是重点，生态宜居是关键，生活富裕是根本。坚持农业农村优先发展，高质量做好“三农”工作，奋力打造新时代乡村振兴的石家庄样板。

持续加快农业转型升级。聚焦打造“四个农业”，深入实施农业供给侧结构性改革三年行动计划，提升农业综合生产能力。推动藏粮于地、藏粮于技，粮食总产稳定在430万吨以上，确保粮食安全。加快推进强筋麦、鲜食玉米产业化，中药材、食用菌基地化，果品、蔬菜品牌化，大力推进奶业振兴，支持君乐宝乳业集团、河北三元做大做强。实施现代农业精品园区建设工程，年内建成市级以上精品园区14家。推进智慧农业延伸应用，建设现代农业园区物联网示范点。完善农产品质量安全追溯体系，强化农产品质量安全监管。毫不松懈抓好非洲猪瘟等畜禽疫病防控工作。大力培育家庭农场、农民合作社等新型经营主体，发展多种形式适度规模经营，构建现代农业发展大格局。

持续深化农村综合改革。以“三块地”为平台，撬动社会资本，让城市资本下乡，解决乡村振兴资本短缺问题，推进城乡一体化和新型城乡结构再造。加快承包地确权登记颁证成果在土地流转、抵押担保、惠农政策落实等方面有效应用，推进农村宅基地“三权分置”制度改革，扎实稳妥开展农村集体经营性建设用地入市试点。创新农业经营方式，完善农业支持保护制度，推进农村集体产权制度改革，年内完成所有村的改革任务。

持续推进农村人居环境整治。围绕生活垃圾处理、污水治理、厕所革命、容貌提升等重点工作，发动群众搞清洁、搞绿化、搞建设、搞管护，形成持续推进机制。稳步推进“空心村”治理，推动人口向城镇集中、劳动力向园区转移、土地向新型经营主体集聚，实施农村新型社区、产业园区、生态功能区“三区同建”。

持续打好精准脱贫攻坚战。围绕“两不愁三保障”，统筹抓好产业扶贫、科技扶贫、就业扶贫、易地扶贫搬迁。实施贫困“清零”行动，确保剩余贫困村全部出列、贫困人口全部稳定脱贫。建立健全防返贫机制，对收入水平略高于建档立卡贫困户的群体，研究制定支持政策。通过建设农村公共基础设施，让非贫困户共享脱贫攻坚成果红利。严格扶贫资金审计，严肃查处扶贫领域存在的违规问题。坚持扶贫与扶志扶智相结合，培育新型职业农民，稳定增加农民收入。

（八）坚定不移打好污染防治攻坚战，强力推进生态环境建设取得新成效。良好的生态环境是最普惠的民生福祉。认真落实习近平生态文明思想，坚守阵地、巩固成果，精准治标、深入治本，推动生态环境持续好转。

坚决打赢蓝天保卫战。围绕大气质量持续改善，加快调整能源结构、产业结构、交通运输结构。加强污染源管控，强力推进农村地区清洁取暖，力争完成剩余“双代”任务，暂不具备替代条件的地区推广使用洁净型煤；坚决防止已淘汰的燃煤锅炉死灰复燃。持续治理“散乱污”企业，深化涉VOCS重点行业整治。加快华荣制药、光华药业等企业退城搬迁，确保2020年前完成石钢搬迁。科学实施重污染天气应急管理各项措施，禁止“一刀切”。继续开展建筑施工扬尘治理，实现视频监控和PM10在线监测“双覆盖”，全面解决黄土裸露问题。统筹“油、路、车”治理，重点整治管控柴油货车、渣土车辆污染，大力推广使用新能源汽车。

坚决打赢碧水、净土保卫战。制定水资源涵养利用规划，抓好地下水超采综合治理。深入实施水污染防治计划，继续开展城市黑臭水体治理、饮用水水源地保护等专项行动。开展重点河流沿线整治，提标改造污水处理厂。加快滹沱河生态修复工程建设，确保8月底一期工程全部完成。加强农业面源污染治理，开展土壤污染状况调查，突出抓好重金属、危险废物和土壤三类污染物防控，有序推进土壤污染治理与修复试点示范。全面落实“生态红线”，实施自然保护区整治行动，严禁各类违规开发活动。继续强力推进造林绿化重点工程，全年完成造林70万亩，完成61处矿山复绿。

坚决保持监管执法高压态势。建立完善县、乡、村网格化环境监管体系，发挥基层环保所（站）作用，充分运用卫星遥感、无人机、物联网等科技手段，建成天、地、空环境立体监管指挥系统。加快建立环境违法企业清单、处罚清单、案件查办清单、追责问责清单，对中央和省环保督察反馈的意见拉条挂账、立行立改。加强对环境监测和运维机构的监管，严厉打击第三方监测数据造假、在线监控数据造假、环境空气质量数据造假等知法犯法、故意损害生态环境问题。

（九）坚定不移保障改善民生，让人民群众共享高质量发展成果。保障改善民生没有终点，只有连续不断的新起点。始终把人民放在心中最高位置，时刻关心老百姓的安危冷暖，完善制度、守住底线，精心做好各项民生工作。

就业是民生之本，牵动着千家万户的生活。落实就业优先政策，聚焦高校毕业生、农民工、下岗转岗职工、退役军人等重点群体，加大就业帮扶和技能培训力度，稳住就业基本盘，全年新增城镇就业12万人。

社保是民生之依。深入推进全民参保计划，推动实现法定人群全面覆盖、待遇水平合理增长。增强救助保障能力，大力发展公益慈善事业。提高城乡居民低保标准和基本医保财政补助标准，扎实做好托底工作。

百年大计，教育为本。继续扩充学前公益普惠资源，促进县域内城乡义务教育一体化发展，着力解决幼儿园“小学化”、义务教育“择校热”等突出问题，巩固提升大班额治理效果。推动普通高中优质、特色发展，继续深化普职融通改革。完成职教园区二期工程，谋划推进高教园区建设。加强京津石教育交流合作，努力培养高素质、专业化、创新型校长教师队伍。

悠悠民生，健康为大。持续深化医药卫生体制改革，推进1-2个城市医疗集团试点工作，支持高邑县创建全省首家紧密型县域医共体，市县公立医院全部实现诊疗信息互通共享。抓好市人民医院赵卜口院区建设。深入推进国家中医药综合改革试验市建设，年内3个县（市、区）创成“河北中医药强县”。完善养老服务体系，推进医养结合，高标准打造全省首家安宁疗护中心，社区养老中心医疗服务覆盖率达到90%。积极承办重大体育赛事，创建全民运动健身模范市。

文化是一座城市的独特印记，是城市最好的底色。加快市、县、乡、村四级公共文化服务体系建设，免费开放公益文化场馆。加大文化惠民卡发放的支持力度，继续开展送戏下基层、全民阅读等品牌文化活动。做好电视剧《白毛女》《故国中山传奇》等文艺创作，擦亮石家庄文化品牌。加强遗址保护利用工作，推动正定古城保护、中山古城考古遗址公园、行唐故郡考古实验室项目建设。

社会安定和谐是人民群众共同的期盼。传承发展新时代“枫桥经验”，提升基层社会治理现代化水平。学习宣传高瑞奎同志先进事迹，弘扬社会正能量。深化“扫黑除恶”专项斗争，围绕创建“无黑”省会城市目标，实施“一案三查”等三大战役，深挖一批大案要案，严惩一批黑恶头目，严查一批背后的“关系网”“保护伞”，还人民群众以安宁。高度重视食品安全工作，强化全链条、全过程监管，全力维护老百姓“舌尖上的安全”。加强应急体系建设，提升防灾减灾救灾能力。落实安全生产责任制，完善安全监管机制，坚决遏制重特大生产安全事故发生。加强市场价格监管，保持物价水平总体稳定。

设身处地解民忧，盈科而进办实事。今年我们将继续办好惠民利民十件实事。①完成200个老旧小区改造。②打通育才街等城区10条“断头路”。③新创建30所普惠性幼儿园。④启动建设市儿童医院（市妇幼保健院）。⑤实行恶性肿瘤、白血病、慢性肾衰竭等7种门诊特殊病零门槛报销，减轻群众就医负担。⑥新建和升级改造25家便民市场。⑦新建15万平方米街旁游园。⑧建设生活垃圾分类样板小区、单位、学校各100个。⑨对1000户贫困重度残疾人家庭进行无障碍改造。⑩完成25万座农村户用厕所改造。

加强全民国防教育、国防动员和后备力量建设，强化退役军人服务保障，维护军人军属合法权益，让军人成为全社会尊崇的职业。充分发挥工会、共青团、妇联等人民团体桥梁纽带作用。继续做好民族宗教、新闻出版广电、外事侨务、人民防空、史志档案、气象地震、妇女儿童、老龄、残疾人等工作，开展经济普查和国土调查，促进各项社会事业全面进步。

各位代表，大道至简，实干为要。我们将以抓铁有痕、踏石留印的干劲，撸起袖子加油干，全力以赴完成各项任务，绝不辜负全市人民的期望和重托。

四、全面加强政府自身建设

新时代、新要求，我们必须始终把发展的重任扛在肩上，把群众的利益放在心上，努力建设科学规范、运转协调、廉洁高效、人民满意的服务型政府。

（一）坚持政治引领，提高政治站位。自觉以习近平新时代中国特色社会主义思想为指引，增强“四个意识”，坚定“四个自信”，做到“两个维护”，始终与党中央保持高度一致，牢记初心使命，忠诚于党，忠诚于人民，确保各项工作始终沿着正确的政治方向前进。

（二）坚持依法行政，规范权力运行。强化法治思维，坚持依法行政，规范执法行为，完善决策机制，努力提高依法决策水平。自觉接受人大法律监督、工作监督和政协民主监督，自觉接受人民群众监督和社会舆论监督，让权力在阳光下运行，确保人民赋予的权力始终为人民服务、为人民谋利益。

（三）坚持务实担当，改进工作作风。功成不必在我、功成必定有我，大力开展“效能革命”，力戒形式主义、官僚主义，坚决惩治庸政懒政怠政行为，始终保持工作激情，促进干部作风实现根本转变。持续深化“双创双服”“双问计”活动，以干事创业的积极成效取信于民、造福于民。

（四）坚持清正廉洁，树立良好形象。以永远在路上的韧劲和执着，深入推进政府系统党风廉政建设和反腐败斗争，持之以恒正风肃纪，严格执行八项规定及实施细则，严格落实廉洁自律准则，坚守底线、远离红线、不碰高压线，真正做到干部清正、政府清廉、政治清明。

各位代表！扬帆新时代，奋斗正当时。让我们更加紧密地团结在以习近平同志为核心的党中央周围，在省委、省政府和市委的正确领导下，锐意进取，扎实工作，为建设现代省会、经济强市作出新的更大贡献，以优异成绩庆祝中华人民共和国成立70周年！
